# Sinus pericranii presenting with macrocephaly and mental retardation

**DOI:** 10.4103/1817-1745.66665

**Published:** 2010

**Authors:** R. B. Kamble, N. K. Venkataramana, L. Naik, Shailesh, R. Shetty

**Affiliations:** Department of Radiology and Neurosurgery, BGS Global Hospital, Uttarahalli Road, Kengeri, Bangalore - 560 098, India

**Keywords:** Digital subtraction angiography, macrocephaly, magnetic resonance imaging, sinus pericranii

## Abstract

We present a rare case of right parietal sinus pericranii in a 2-year-old female child who presented with a compressible swelling on the right side of the scalp since 3 months of age, with a large head. Magnetic resonance imaging along with venography and conventional angiogram was performed, which confirmed the diagnosis.

## Introduction

Sinus pericranii is a rare congenital vascular anomaly, which is a fluctuating, compressible venous scalp mass that connects directly to the intracranial dural sinuses through dilated diploic and emissary veins.[1] This anomaly can be readily diagnosed by radiological investigations. Findings on imaging, like magnetic resonance (MR) venography, have already been described.[2] We report a case of sinus pericranii in a 2-year-old female child with a unique presentation of scalp swelling, macrocephaly and mental retardation along with MR imaging (MRI), MR venography and conventional angiographic findings.

## Case Report

**Figure 1:**
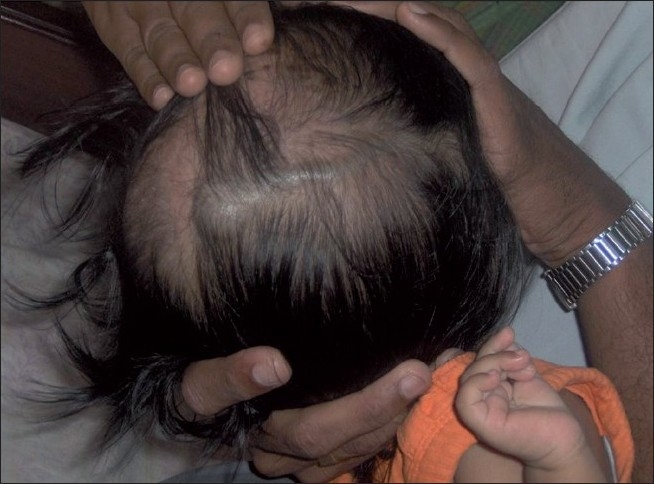
Large bluish dilated vein on the scalp with venous sac in the midline

**Figure 2:**
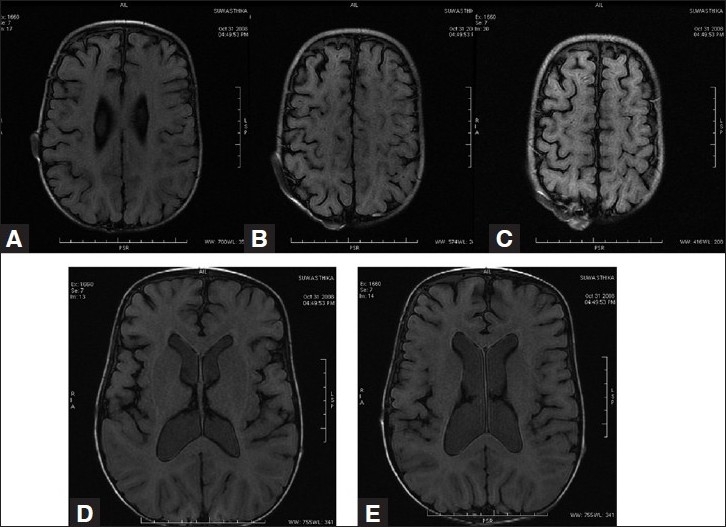
(A–C) T1-weighted images show the hypointense scalp vein with bony defect and venous sac. (D, E) The dilated ventricular system

**Figure 3:**
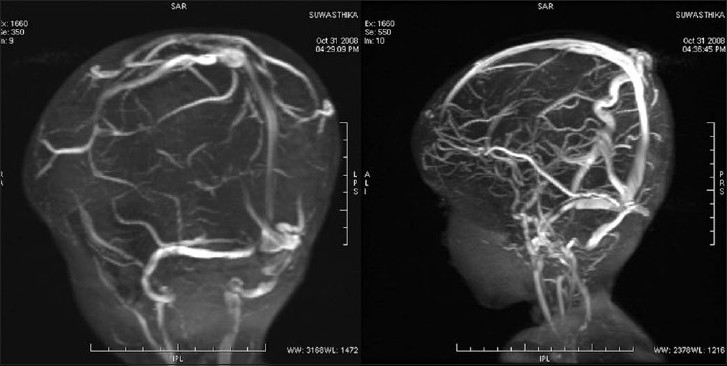
Magnetic resonance venography showing a large extracranial vein with venous sac in the midline communicating with the superior sagittal sinus

**Figure 4:**
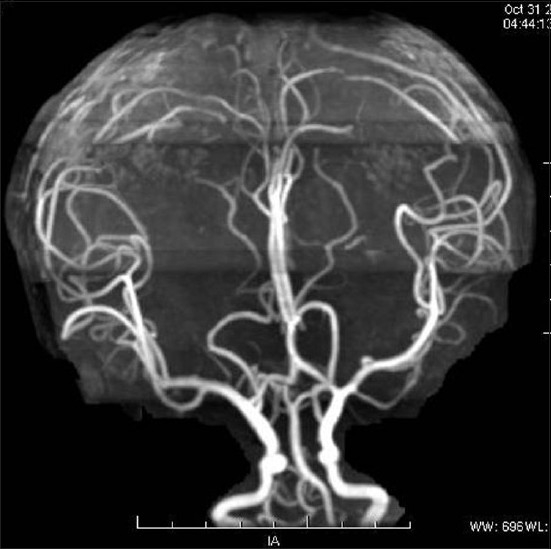
Magnetic resonance angiography shows a normal arterial tree

**Figure 5:**
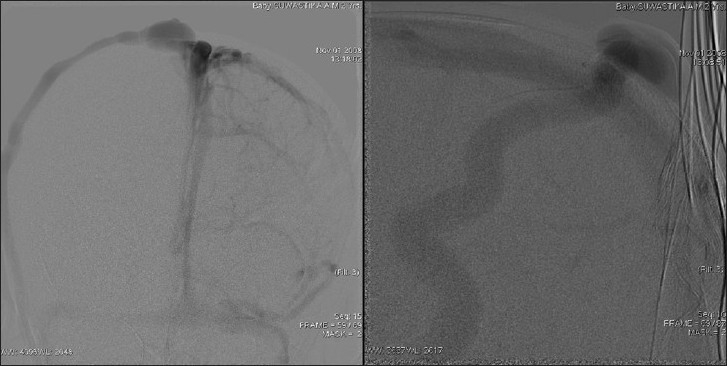
Digital subtraction angiography in the late venous phase showing the large extracranial vein communicating with the superior sagittal sinus through the emissary vein

This 2-year-old girl child born to nonconsanguineous parents presented with a history of large head and an abnormal compressible swelling on the right side of the scalp when she was 3 months old [Figure 1]. The swelling increased in size when the child cried. On examination, the child was found to have macrocephaly with delayed milestone in the form of neck holding at 1 year and standing with support at 22 months. On physical examination, there was a large compressible bluish scalp mass on the right parietal region with bony dents underneath. The mass was seen to increase in size on crying. The anterior and posterior fontanelles were open. The child underwent MRI along with MR venography and digital subtraction angiography (DSA) to confirm diagnosis. MRI showed a T1-hypointense swelling in the right parietal region with bony defect communicating with the superior sagittal sinus and ventricular dilatation [Figure 2]. MR venography showed a dilated large extracranial vein on the right parietal region communicating with the superior sagittal sinus through dilated diploic and emissary veins [Figure 3]. MR angiography was normal [Figure 4]. Subsequently, the child underwent DSA under general anesthesia, which confirmed large extracranial scalp vein draining into the superior sagittal sinus seen on the venous phase. Bilateral internal and external carotid arteries were normal. No dural arteriovenous fistula was found [Figure 5]. Thus, the diagnosis of sinus pericranii was made depending on MR and DSA findings. The child was kept on follow-up.

Sinus pericranii is a rare congenital venous anomaly, which is soft and compressible scalp mass that connects directly to the intracranial dural sinuses through dilated diploic and emissary veins. They are known to increase in size on valsalva manoeuvre or raised intracranial pressures and reduce on nondependent positions.[1] Mainly congenital, traumatic and spontaneous causes are described for the development of sinus pericranii. Traumatic cause is mainly due to tearing of emissary veins and later developing into communicating blood cyst.[1] Pathologically, they can be termed congenital if lined by endothelium.[3] Sinus pericranii can appear at any age, usually <30 years, common in males and, although usually asymptomatic, may present with nausea, vomiting and vertigo.[2,4] Differential diagnosis for sinus pericranii includes dural fistula, arteriovenous malformation and other midline masses of scalp-like dermoid, lipoma and encephalocele.[4] Sometimes, it can mimic subepicranial varix where there is dilated venous sac on the scalp without communication with intracranial dural sinuses.[5] Sinus pericranii may be associated with various other anomalies, like systemic angiomas and craniosynostosis.[6,7] It can be diagnosed by clinical examination and radiological imaging. Usefulness of MR venography and computed tomography angiography has already been described to confidently diagnose sinus pericranii and exclude other mimicking causes of scalp swelling. However, conventional angiography may still be useful to rule out other vascular malformations like dural fistula and arteriovenous malformation. Demonstration of extracranial venous sac communicating with intracranial dural sinus via diploic or emissary veins is necessary for the diagnosis of sinus pericranii, which can be easily performed by today’s imaging modalities.[2,5]

The present case showed a large head with developmental delay. Imaging showed mild hydrocephalus. Most of the cases described in the literature are associated with craniosynostosis; however, our case showed macrocephaly. Presence of macrocephaly may be due to hydrocephalus. Developmental delay may be attributed to increased venous pressure in the dural sinuses causing raised intracranial pressures leading to delayed development. Development of transient venous hypertension by sinus pericranii has been described previously.[8]

Treatment options in symptomatic patients include surgical resection or a transvenous endovascular approach. If treatment has to be contemplated, then Gondolfo *et al*. have recommended to assess the drainage pattern of sinus pericranii. If sinus pericranii is dominant, *i.e*. if drainage of the brain is through sinus pericranii bypassing the usual venous outlets, then treatment should be avoided.[9] However, spontaneous regression of sinus pericranii has also been reported.[10] Our case was kept on follow-up under close observation to be treated later if the clinical condition worsened.

## Conclusion

Sinus pericranii can also be associated with macrocephaly and developmental delay due to possible venous hypertension, which was not described before.
